# Effects of nitrogen levels on gene expression and amino acid metabolism in Welsh onion

**DOI:** 10.1186/s12864-021-08130-y

**Published:** 2021-11-07

**Authors:** Chen Zhao, Guanchu Ma, Lin Zhou, Song Zhang, Le Su, Xin Sun, Orlando Borrás-Hidalgo, Kunlun Li, Qiulin Yue, Lin Zhao

**Affiliations:** 1 State Key Laboratory of Biobased Material and Green Papermaking, Shandong Provincial Key Lab. of Microbial Engineering, Qilu University of Technology (Shandong Academy of Sciences), Jinan, China; 2 Shandong Provincial Key Laboratory of Food and Fermentation Engineering, Shandong Food Ferment Industry Research & Design Institute, Qilu University of Technology (Shandong Academy of Sciences), Jinan, China; 3 Jinan Hangchen Biotechnology Co., Ltd, Jinan, China; 4 School of Life Sciences, Anhui Agricultural University, Hefei, China

**Keywords:** Welsh onion, Nitrogen, Transcriptome, Amino acids

## Abstract

**Background:**

Welsh onion constitutes an important crop due to its benefits in traditional medicine. Nitrogen is an important nutrient for plant growth and yield; however, little is known about its influence on the mechanisms of Welsh onion regulation genes. In this study, we introduced a gene expression and amino acid analysis of Welsh onion treated with different concentrations of nitrogen (N0, N1, and N2 at 0 kg/ha, 130 kg/ha, and 260 kg/ha, respectively).

**Results:**

Approximately 1,665 genes were differentially regulated with different concentrations of nitrogen. Gene ontology enrichment analysis revealed that the genes involved in metabolic processes, protein biosynthesis, and transportation of amino acids were highly represented. KEGG analysis indicated that the pathways were related to amino acid metabolism, cysteine, beta-alanine, arginine, proline, and glutathione. Differential gene expression in response to varying nitrogen concentrations resulted in different amino acid content. A close relationship between gene expression and the content of amino acids was observed.

**Conclusions:**

This work examined the effects of nitrogen on gene expression and amino acid synthesis and provides important evidence on the efficient use of nitrogen in Welsh onion.

## Background

Nitrogen (N) is considered one of the most important nutrients required for plant growth and yield [1, 2]; therefore, crop yield and productivity have a strong relationship with the supply of nitrogen. The demand is fulfilled by the application of nitrogen fertilizers in the field, which come in various chemical forms, such as inorganic NO_3_^−^, NH_4_^+^, and organic urea, which is the most commonly applied nitrogen fertilizer worldwide [3]. However, excessive use of urea increases production costs and environmental pollution. Therefore, increasing nitrogen use efficiency (NUE) is important for sustainable agriculture.

Welsh onion (*Allium fistulosum L.*) is an important economical crop widely cultivated throughout the world, particularly in Asian countries [4]. It is often used as an ingredient for its special flavor and aroma and is considered a good source of nutrition; it is also used in traditional medicine [5]. However, despite its nutritional and medicinal value, information about this non-model plant’s response to nitrogen is limited.

NUE in plants is highly complex and can induce diverse processes at both physiological and molecular levels. Ribonucleic acid-sequencing (RNA-seq) technology is a powerful tool that has been widely used to quantify gene expression levels in biological studies. RNA-seq was successfully applied in discovering key genes in populous [6], cucumber [7], *Arabidopsis thaliana*, and wheat [8, 9]. It was also used in genomics studies on *A. fistulosum* [5, 9], where the gene expression of different varieties of *A. fistulosum* was used [10]. To date, transcriptome studies have been carried out on many crops after nitrogen treatment, including *Arabidopsis* [11, 12], maize [13], poplar [14], and cucumber [7].

Amino acids are important nitrogen storage compounds in plants [15]. As the biosynthesis of amino acids requires nitrogen and carbon elements, nitrogen nutrition [16] and photosynthesis [17] are crucial, and after the uptake of nitrogen, glutamate synthase (GOGAT) and glutamine synthetase (GS) play vital roles in nitrogen assimilation in plants [18]. The relationship between specific genes and amino acids has been reported in other crops [19]. The abundance of nitrogen can strongly affect the biosynthesis of amino acids of tea plants, thus influence tea quality [20]. However, little information is available regarding the metabolism of nitrogen and amino acids and the gene regulation network in *A. fistulosum*.

In this study, we introduced differentially expressed gene (DEG) regulations and amino acids to investigate the relationship between nitrogen supply and metabolism in Welsh onion. We used RNA-seq technology and measured amino acids to explore the gene regulation network in *A. fistulosum*. There was a close relationship between gene expression and the content of amino acids; therefore, specific DEGs might improve the understanding of the influence of nitrogen in Welsh onion.

## Results

### Sequencing and de novo assembly

To study the global transcriptional response of *A. fistulosum* to various urea concentrations (N0, N1, and N2), we analyzed samples subjected to various nitrogen treatments using RNA-seq. De novo assembly was performed using a Trinity assembler, and the length distributions of the contigs, transcripts and unigenes are shown in Table 1. The next generation short-read sequences were assembled into 536,449 transcripts with an average length of 822.26 bp. The transcripts were subjected to cluster and assembly analyses. The longest transcript was taken as the sample unigene for data. A total of 247,703 unigenes with an average length of 634.16 bp were obtained. The N50 values of the transcripts and unigenes were 1,343 and 948 bp, respectively. The GC content of Welsh onion unigenes was 37.62 %.

**Table 1:** Summary of sequence assembly for *A. fistulosum*

	Contig	Transcript	Unigene
Total length (bp)	215,015,755	441,101,605	157,084,336
Sequence number	703,130	536,449	247,703
Max. length (bp)	15,342	15,675	15,675
Mean length (bp)	305.80	822.26	634.16
N50 (bp)	424	1343	948
N50 sequence no.	107,314	94,254	41,994
GC%	37.72	37.47	37.62

### Functional annotation and classification

All assembled unigenes were subjected to BLASTx similarity analysis with an E-value of 10^−5^ against different NCBI databases, including NR, Gene Ontology (GO), the Kyoto Encyclopedia of Genes and Genomes (KEGG), evolutionary genealogy of genes: Non-supervised Orthologous Groups (eggNOG), and Swiss-Prot. As shown in Table 2, there were 59,689 unigenes (24.1 %) annotated in the NR database, 22,292 (9 %) annotated in the GO database, 5,901 (2.38 %) annotated in the KEGG database, 56,192 (22.69 %) annotated in the eggNOG database, and 47,305 (19.1 %) matched in the Swiss-Prot database.

**Table 2:** Functional annotation of *A. fistulosum* transcriptome

Database	Number	Percentage
NR GO KEGG eggNOG Swiss-Prot All databases	59,689 22,292 5,901 56,192 47,305 3,260	24.10 9.00 2.38 22.69 19.10 1.32

### Differentially expressed genes in response to nitrogen

**Fig. 1:**
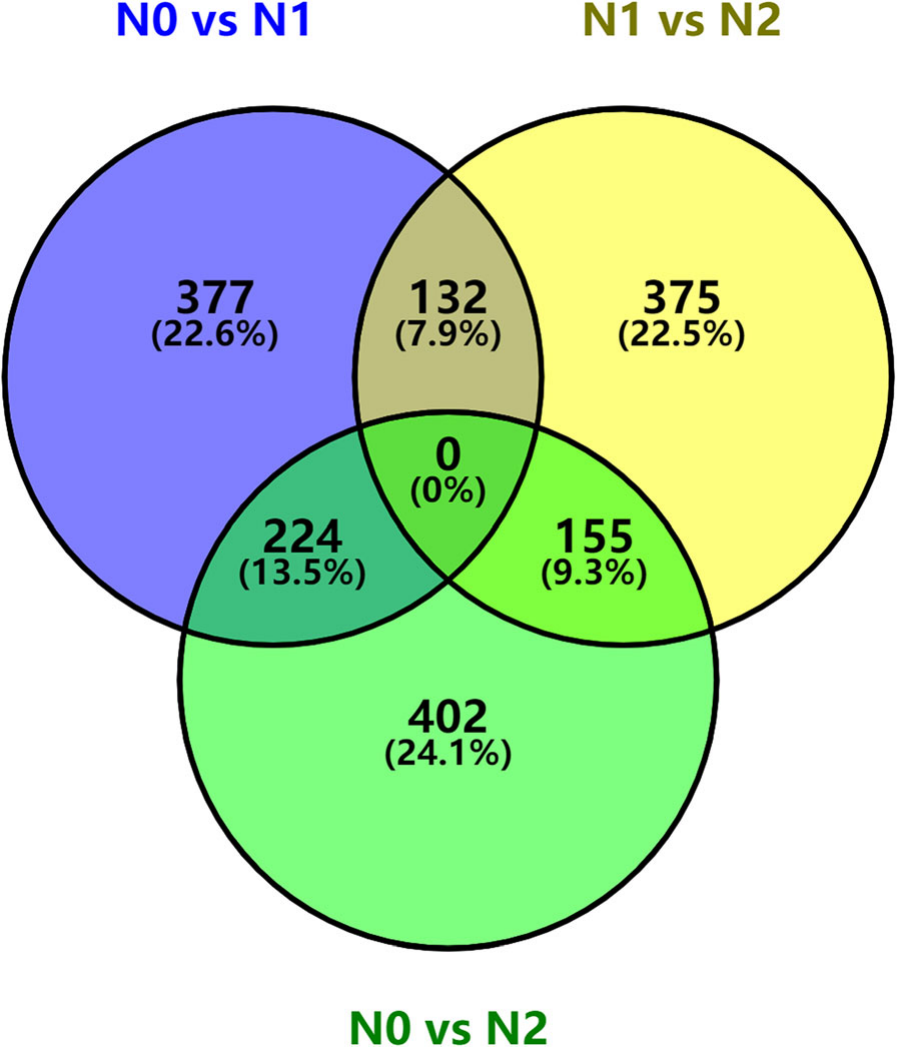
Venn diagram showing differentially expressed genes after nitrogen treatment: N0: nitrogen-free; N1: half nitrogen; N2: full nitrogen

Table 3 compares plants without nitrogen (N0), 369/364 DEGs were up/down-regulated in the Welsh onion treated with half-levels of nitrogen (N1), 414/367 DEGs were up/down-regulated after full levels of nitrogen treatment (N2), and 387/275 DEGs were up/down-regulated in the pseudostems after full levels of nitrogen treatment with N1 as the control. As shown by the Venn diagram (Figs. 1), 224 DEGs overlapped in N1 vs. N0 and N2 vs. N0.

**Table 3:** Differentially expressed genes after nitrogen treatment

Control	Case	Up-regulated genes	Down-regulated genes	Total DEGs
N0	N1	369	364	733
N0	N2	414	367	781
N1	N2	387	275	662

**Fig. 2:**
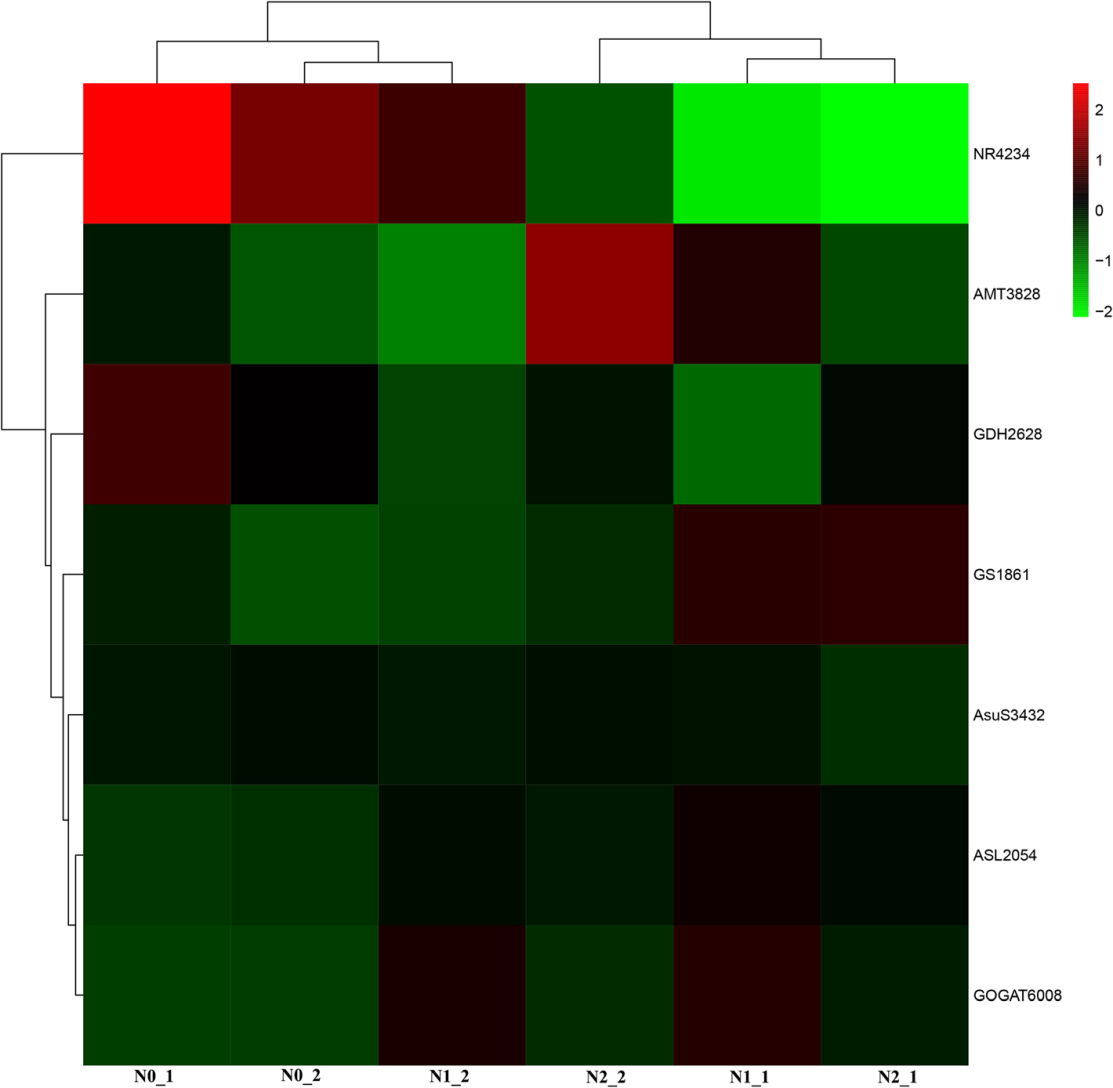
Heatmap of the DEGs (FPKM) of Welsh onion after treatment with different concentrations of nitrogen: *N0*: nitrogen-free; *N1*: half nitrogen; *N2*: full nitrogen; *AsuS*: argininosuccinate synthase; *ASL*: argininosuccinate lyase; *NR*: nitrate reductase; *AMT*: ammonium transporter; *GDH*: glutamate dehydrogenase; *GOGAT*: glutamate synthase; *GS*: glutamine synthetase

Seven genes were identified involving in N-uptake or assimilation from the transcriptome data. The lowest expression level of nitrate reductase (NR) and the highest expression level of GS corresponded to plants treated with the highest nitrogen concentration (Fig. 2). Down-regulation was observed for argininosuccinate synthase (AsuS) in all the groups. The order of the Welsh onion, according to the expression level of argininosuccinate lyase (ASL) and GOGAT, was N1>N2>N0, whereas the order for glutamate dehydrogenase (GDH) was the opposite (N1<N2<N0). The expressions of ammonium transporter (AMT) were not differential in any of the groups in response to nitrogen treatment.

### GO and KEGG enrichment analysis

The DEGs in comparative conditions were subjected to GO enrichment analysis to predict the biological functions of candidate genes in response to nitrogen. These transcripts were further classified into three major categories, but most of the assignments belonged to biological processes and molecular function. Significant GO terms were selected with a cut-off *P*-value of 0.05. Only one term (GO:0004766) belonging to molecular function was obtained between nitrogen-free and full levels of nitrogen treatment (data not shown). Six GO terms were classified as molecular function, and 12 GO terms were classified as biological processes (Tables 4 and 5) in the other two comparable groups. Among them, six terms were related to the biosynthesis of peptides or amino acids (GO:0004766, GO:0006412, GO:0043043, GO:0043604, GO:0006518, GO:0043603), while one term was related to the biosynthesis of nitrogen compound (GO:0044271). Four terms were related to transporter activity (GO:0080161, GO:0010329, GO:0015562, GO:0009926). These results indicated that candidate genes were closely related to the metabolism, biosynthesis, and transportation of nitrogen, and especially with amino acid metabolism.

**Table 4:** GO enrichment analysis of the DEGs between nitrogen-free and half nitrogen (*P* < 0.05)

Category	GO_Term	Annotation	Gene ratio	Background ratio	*P*-value
molecular_function	GO:0080161	Auxin transmembrane transporter activity	2/733	4/22,292	0.00267
molecular_function	GO:0010329	Auxin efflux transmembrane transporter activity	2/733	4/22,292	0.00267
molecular_function	GO:0015562	Efflux transmembrane transporter activity	2/733	5/22,292	0.00445
biological_process	GO:0010252	Auxin homeostasis	2/733	4/22,292	0.00658
biological_process	GO:0010817	Regulation of hormone levels	3/733	38/22,292	0.02225
biological_process	GO:0009926	Auxin polar transport	2/733	10/22,292	0.04891

**Table 5:** GO enrichment analysis of the DEGs between half nitrogen and full levels of nitrogen (*P* < 0.05)

Category	GO_Term	Annotation	Gene ratio	Background ratio	*P*-value
molecular_function	GO:0004766	Spermidine synthase activity	16/662	1,154/22,292	0.00848
molecular_function	GO:0005198	Structural molecule activity	15/662	1,027/22,292	0.0000295
molecular_function	GO:0003735	Structural constituent of ribosome	20/662	2,310/22,292	0.0000373
biological_process	GO:0044271	Cellular nitrogen compound biosynthetic process	15/662	1,506/22,292	0.00241
biological_process	GO:0006412	Translation	15/662	1,512/22,292	0.00683
biological_process	GO:0043043	Peptide biosynthetic process	2/662	6/22,292	0.00715
biological_process	GO:0043604	Amide biosynthetic process	15/662	1,555/22,292	0.00981
biological_process	GO:0006518	Peptide metabolic process	15/662	1,569/22,292	0.01084
biological_process	GO:0010467	Gene expression	18/662	2,186/22,292	0.0133
biological_process	GO:0043603	Cellular amide metabolic process	15/662	1,654/22,292	0.01946
biological_process	GO:0034645	Cellular macromolecule biosynthetic process	18/662	2,282/22,292	0.02289
biological_process	GO:0009059	Macromolecule biosynthetic process	18/662	2,313/22,292	0.02708

KEGG pathway enrichment analysis was performed to categorize the biochemical pathways of DEGs. To better understand the biological functions and pathways of candidate genes in the Welsh onion with different N-supplements, all of the DEGs were annotated in the KEGG database. The pathways with *P*-value < 0.05 were regarded as significant. In terms of the enrichment analysis of the DEGs between nitrogen-free and half nitrogen treatment, two pathways (ko00190 and ko00904) were detected, which related to oxidative phosphorylation and diterpenoid biosynthesis, respectively (data not shown). Our results revealed that 21 pathways were involved after nitrogen treatment (Tables 6 and 7), with four similar pathways among different nitrogen concentrations (ko00270, ko00410, ko00330, ko00480). Interestingly, all of these pathways were related to amino acid metabolism. All the findings indicated that the level of nitrogen can affect the biosynthesis of amino acids.

**Table 6:** KEGG pathway enrichment analysis of the DEGs between nitrogen-free and full levels of nitrogen treatment (*P* < 0.05)

Pathway_ID	Pathway	Annotation	Gene ratio	Background ratio	*P*-value
ko00904	Diterpenoid biosynthesis	Metabolism of terpenoids and polyketides	2/781	11/5,901	0.0003738
ko00270	Cysteine and methionine metabolism	Amino acid metabolism	3/781	55/5,901	0.0003938
ko00410	Beta-alanine metabolism	Metabolism of other amino acids	2/781	22/5,901	0.0015428
ko04066	HIF-1 signaling pathway	Signal transduction	2/781	29/5,901	0.0026817
ko00330	Arginine and proline metabolism	Amino acid metabolism	2/781	32/5,901	0.003261
ko00480	Glutathione metabolism	Metabolism of other amino acids	2/781	42/5,901	0.005571
ko01230	Biosynthesis of amino acids	Overview	3/781	150/5,901	0.007069
ko00401	Novobiocin biosynthesis	Biosynthesis of other secondary metabolites	1/781	4/5,901	0.010804
ko00010	Glycolysis/ Gluconeogenesis	Carbohydrate metabolism	2/781	72/5,901	0.015775
ko00905	Brassinosteroid biosynthesis	Metabolism of terpenoids and polyketides	1/781	9/5,901	0.024156
ko03010	Ribosome	Translation	3/781	267/5,901	0.033123
ko00950	Isoquinoline alkaloid biosynthesis	Biosynthesis of other secondary metabolites	1/781	13/5,901	0.034715
ko00960	Tropane, piperidine, and pyridine alkaloid biosynthesis	Biosynthesis of other secondary metabolites	1/781	14/5,901	0.037338

**Table 7:** KEGG pathway enrichment analysis of the DEGs between half nitrogen and full levels of nitrogen (*P* < 0.05)

Pathway_ID	Pathway	Annotation	Gene ratio	Background ratio	*P*-value
ko00270	Cysteine and methionine metabolism	Amino acid metabolism	3/662	22/5,901	0.0005864
ko00410	Beta-alanine metabolism	Metabolism of other amino acids	2/662	32/5,901	0.0012478
ko00330	Arginine and proline metabolism	Amino acid metabolism	2/662	42/5,901	0.0021466
ko00480	Glutathione metabolism	Metabolism of other amino acids	2/662	55/5,901	0.0036590
ko04913	Ovarian steroidogenesis	Endocrine system	1/662	3/5,901	0.0050761
ko03010	Ribosome	Translation	3/662	267/5,901	0.0086727
ko00140	Steroid hormone biosynthesis	Lipid metabolism	1/662	6/5,901	0.0101291
ko00830	Retinol metabolism	Metabolism of cofactors and vitamins	1/662	7/5,901	0.0118082
ko00380	Tryptophan metabolism	Amino acid metabolism	1/662	20/5,901	0.0334053
ko00980	Metabolism of xenobiotics by cytochrome P450	Xenobiotics biodegradation and metabolism	1/662	21/5,901	0.0350490
ko04020	Calcium signaling pathway	Signal transduction	1/662	24/5,901	0.0399647
ko04022	cGMP-PKG signaling pathway	Signal transduction	1/662	30/5,901	0.0497285

### Regulation of amino acid content in response to different concentrations of nitrogen

**Fig. 3:**
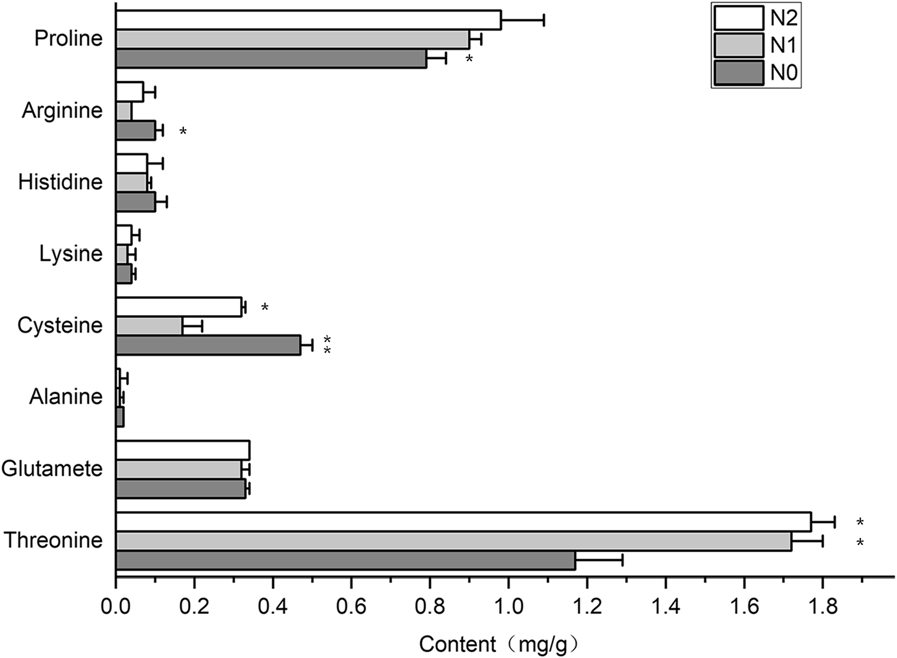
Contents of amino acids (mg/g fresh weight) in pseudostems after nitrogen treatment; significance values were expressed as * *P* < 0.05, ** *P* < 0.01

**Fig. 4:**
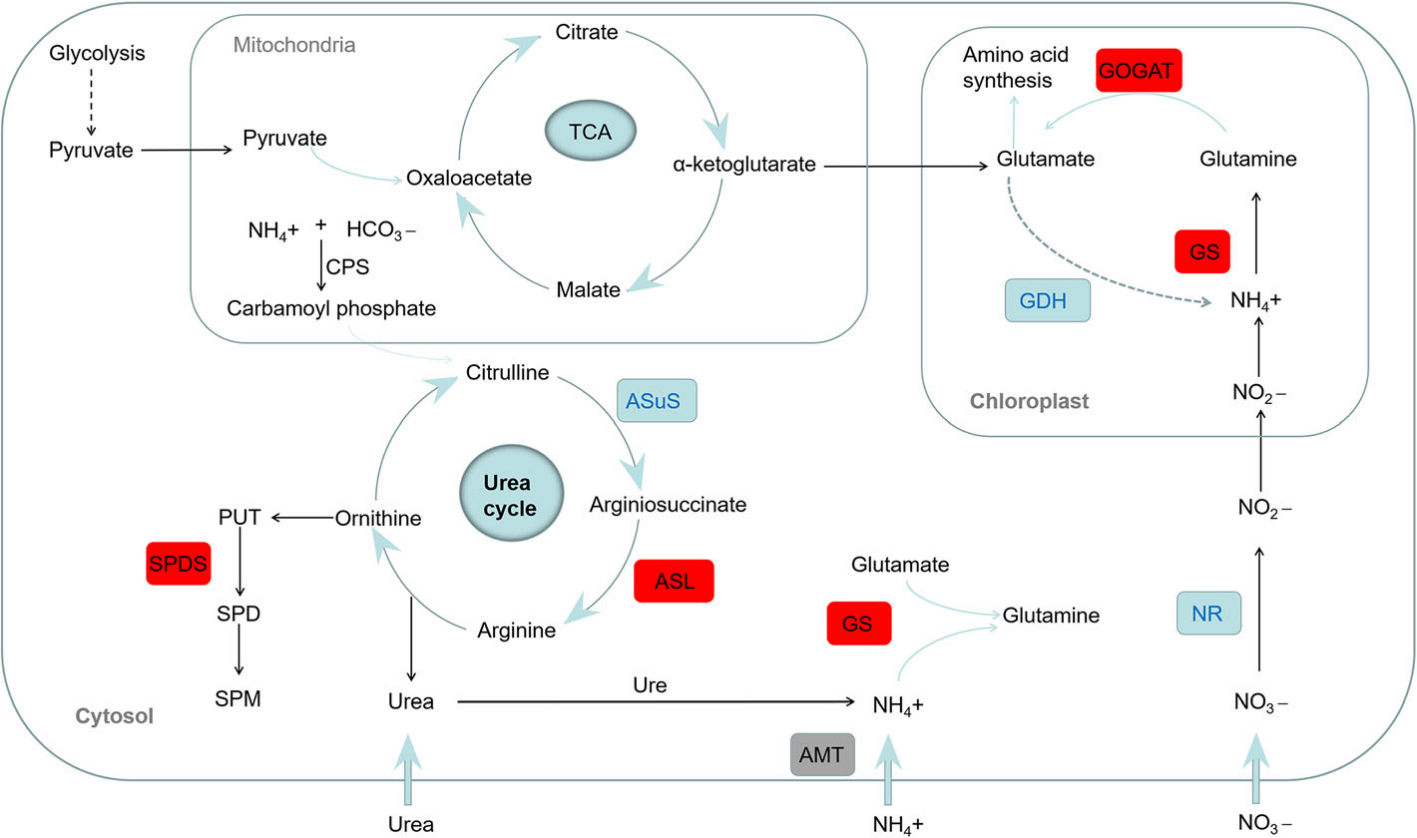
The DEGs of pathways related to nitrogen transport and assimilation: *CPS*: carbamoyl phosphate synthase; *GS*: glutamine synthetase; *GOGAT*: glutamate synthase; *GDH*: glutamate dehydrogenase; *AsuS*: argininosuccinate synthase; *ASL*: argininosuccinate lyase; *Ure*: urease; *PUT*: putrescine; *SPDS*: spermidine synthase; *SPD*: spermidine; *SPM*: spermine; *AMT*: ammonium transporter; *NR*: nitrate reductase; red represents the transcripts positively regulated by urea treatment; blue represents transcripts negatively regulated by urea treatment

After nitrogen treatment, the pseudostems contained significantly more threonine and proline than the nitrogen-free group (Fig. 3). The amounts of two amino acids were similar after different concentrations of nitrogen. The contents of glutamate, alanine, lysine, and histidine did not show marked differences with altered levels of nitrogen. Meanwhile, higher contents of cysteine and arginine were detected in the nitrogen-free samples. Higher contents of cysteine may attribute to the higher expression level of cysteine synthase (CS) corresponded to nitrogen-free group (data not shown). A higher expression level of genes was observed that encoded spermidine synthase involved in the biosynthesis pathway of proline, beta-alanine, cysteine, arginine, and glutathione (Tables 5 and 8). As the precursor of spermidine, decreased level of arginine was detected after nitrogen treatment. Alpha-enolase and glyceraldehyde-3-phosphate dehydrogenase genes were also up-regulated. To show the effects of the candidate genes, we proposed a regulation network of nitrogen metabolism and amino acid synthesis (Fig. 4).

**Table 8:** DEGs involved in the process of amino acids metabolism after nitrogen treatment; the up-gene ID represents the unigenes named in the assembly of *A. fistulosum*

Up-gene ID	KEGG	Swissprot
TRINITY_DN53778_c0_g1	K00815	Nicotianamine aminotransferase A
TRINITY_DN54196_c0_g1	K01689	Alpha-enolase
TRINITY_DN66403_c3_g1	K00134	Glyceraldehyde-3-phosphate dehydrogenase
TRINITY_DN68797_c1_g2	K00797	Spermidine synthase 1
TRINITY_DN73431_c4_g4	K00797	Spermidine synthase 1

## Discussion

Transcriptome analysis is an effective approach in identifying metabolic pathways and novel plant genes without a genomic sequence [21, 22]. The importance of using nitrogen efficiently in plants’ growth and yield has become a very attractive scientific topic. NUE includes the uptake and assimilation of nitrogen into plants, thus, identifying the genes involved in this process is currently a key step. Several genes involved in the use of nitrogen were reported in *Arabidopsis* and rice [15], but although Welsh onion is an important crop, little is known about the role of these types of genes. In this study, RNA-seq was used to investigate the genes involved in NUE in response to different concentrations of nitrogen. Differential expression behavior of genes involved in N-uptake and assimilation and urea cycle were detected in response to nitrogen treatment (Fig. 4). Genes encoding for enzymes of N-assimilation, including NR and GDH, and gene for AsuS of urea cycle were found to be down-regulated. However, genes for GS and GOGAT of N-assimilation, and ASL of urea cycle were up-regulated after nitrogen supply.

Nitrogen uptake includes the assimilation and remobilization of organic nitrogen in the whole plant [23]. Urea had a repressive effect on NO_3_^−^ influx but promoted NH_4_^+^ uptake in *Arabidopsis* [24]. This was consistent with the phenomena introduced in Welsh onion after urea treatment with a down-regulated expression pattern of a nitrogen uptake gene (NR) (Fig. 2). NR is a key enzyme that catalyzes the first step of nitrate assimilation, and its reduced expression under nitrogen stress indicated the regulation of this enzyme in rice [25]. The down-regulated pattern was also detected in nitrogen-deficient physic nut [26]. In this study, NR expression showed a reducing trend related to nitrogen concentration, showing a similar regulation pattern in *A. fistulosum.*

Several works on the correlation between gene expression and the biosynthesis of amino acids under nitrogen supplementation conditions have been conducted on *Arabidopsis* [27], rice [28], and tea [19]. In this study, we identified 1,665 DEGs in the pseudostems of Welsh onion in response to different concentrations of nitrogen. These DEGs provided candidate genes for further analysis of biological function and pathways regarding nitrogen transportation and metabolism. GO enrichment analysis efficiently predicted the biological functions according to the transcriptome data [29]. Multiple GO terms were significantly represented under different levels of nitrogen treatment, especially those for amino acid and peptide biosynthesis, metabolism, and transporters. Moreover, KEGG analysis showed that a substantial number of DEGs were detected under nitrogen starvation and led to significant changes in some metabolic pathways, including amino acid metabolism, translation, carbohydrate metabolism, and biosynthesis of other secondary metabolites. Our previous results indicated that the highest phenolic content was observed in the N2 treatment group, whereas that in the N0 group was the lowest [30]. This can be attributed to the DEGs identified in the pathways of metabolism of terpenoids and polyketides between nitrogen-free and full levels of nitrogen treatment (Table 6).

The results revealed that the pseudostems of the Welsh onion, after nitrogen treatment, contained significantly more threonine and proline than the nitrogen-free group, while a higher content of cysteine and arginine were detected in the nitrogen-free samples. Cysteine and glutathione metabolism are reported to indicate parallels with *Allium* flavour precursor biosynthesis [31]. Moreover, cysteine is the first organic product generated from S [32]. Cysteine synthase is the last enzyme of sulfate assimilation pathway, and O-acetylserine (OAS), the precursor of cysteine, is derived from the carbon and nitrogen assimilation pathways [33]. The down-regulated pattern of CS was detected after nitrogen treatment. For one hand, the synthesis of cysteine decreased; for another, with the precursor of cysteine, flavour compounds were synthesized. The results indicated that nitrogen supply affect sulphur assimilation pathways, including the synthesis of cysteine and other products, thus affacting the flavour of Welsh onion. Ornithine is the point of entry for the biosynthesis of polyamines such as putrescine, spermidine, and spermine, which are used to store excess organic nitrogen in plant tissues [34]. Arginine is the precursor of ornithine, and this was probably the reason why the arginine content was reduced after nitrogen treatment (Fig. 4). The alpha-enolase and glyceraldehyde-3-phosphate dehydrogenase genes were also up-regulated. These are key enzyme genes related to glycolysis, and the catalytic products are the main precursors of amino acids. The number of precursors may determine the difference between the content of amino acids. It seems that nitrogen treatment might also promote glycolysis and tricarboxylic acid cycle (TCA) flux as well as amino acid metabolism. Our previous results showed that the highest yields were detected in the full levels of nitrogen treatment (N2) followed by half-levels of nitrogen (N1) [35]. Therefore, it is possible that nitrogen triggers a number of molecular and physiological events that lead to the increase of plant biomass, especially for carbohydrate metabolism, amino acid metabolism.

In the glutamine–glutamate cycle, GOGAT, GS, and GDH were the key enzymes regulating the amount of each compound. Glutamate was always used as the nitrogen source in the biosynthesis of nitrogen compounds [19]. Under nitrogen starvation, the transcript levels of GS and GOGAT were up-regulated, whereas GDH was found to be significantly down-regulated (Fig. 2). We also measured the metabolites of the glutamine–glutamate cycle and found that the glutamate content did not significantly change after treatment with different concentrations of nitrogen. It can be assumed that the higher expression level of the two genes with nitrogen treatment was induced by urea-derived ammonium through a positive feedback mechanism via the GS–GOGAT cycle.

## Conclusions

This work examined the influence of nitrogen in the activation of nitrogen-related genes and amino acid metabolism in Welsh onion for the first time. The transcriptome analysis of Welsh onion on different concentrations of nitrogen treatment revealed that 1,665 genes were significantly regulated. GO analysis revealed that the DEGs were associated with diverse processes. The metabolism and transporters of various amino acids were highly represented, indicating the processes involved with using nitrogen. KEGG analysis provided the enrichment pathways related to amino acid metabolism. However, the DEGs’ response to nitrogen application resulted in different contents of amino acids. With the introduction of the effect of nitrogen in gene expression and amino acid synthesis, this work provides important evidence of NUE in Welsh onion.

## Methods

### Plant materials and RNA extraction

The *A. fistulosum* species was obtained from the Shandong Academy of Agricultural Sciences. Planting was performed as previously described [35], and seedlings were grown under field conditions in Zhangqiu district, Jinan city, Shandong Province, China. The soil type was classified as cinnamon soil and had a nitrogen concentration of 80.67 mg kg^−1^ before the experiment. The plants were treated with three different concentrations of urea, namely, nitrogen-free (without urea), half levels of nitrogen (130 kg ha^−1^), and full levels of nitrogen (260 kg ha^−1^). The treatments (0 N, half N, full N) were named N0, N1, and N2. The seedlings were grown in 15 independent plots, with five plots for each nitrogen concentration. The fertilizer was used four times during the growth stages of Welsh onion, on June 25, August 14, August 27, and September 9, 2017, respectively. Welsh onion was collected on October 1, 2017, and further analyses were performed. Of the five field plot replications, two plot replications were randomly selected for each group to make biological replications for RNA extraction. Randomly selected samples of pseudostems in the same plot were pooled together, immediately frozen in liquid nitrogen, and stored at -80 °C. Total RNA was extracted from the pseudostems using Trizol reagent (Invitrogen Life Technologies) following the manufacturers’ instructions. RNA quality was determined using the Agilent 2100 Bioanalyzer (Santa Clara, CA, USA).

### Transcriptome sequencing

For each treatment, three micrograms of RNA of the tissues with different concentrations of nitrogen were used to obtain the cDNA libraries. Sequencing libraries were generated using the TruSeq RNA Sample Preparation Kit (Illumina, San Diego, CA, USA). To select the preferred cDNA fragments that were 200 bp in length, the library fragments were purified using the AMPure XP system (Beckman Coulter, Beverly, CA, USA). DNA fragments with ligated adaptor molecules on both ends were selectively enriched with an Illumina PCR Primer Cocktail in a 15-cycle polymerase chain reaction (PCR) reaction. The 150 bp paired-end cDNA libraries were sequenced on Illumina’s Hiseq 2500 (Shanghai Personal Biotechnology Cp. Ltd.) following standard Illumina methods.

### Functional annotation

To obtain high-quality reads, the raw reads were filtrated to remove low-quality reads and reads with adapters. The reads were assembled using Trinity software with default parameters [36, 37]. First, contigs were obtained by extension based on the overlap between sequences. Next, the contigs were joined into transcripts by paired-end mapping. Finally, the contigs were connected to get sequences that could not be extended at either end. The longest transcripts of each gene in the upper genome were extracted as the reference transcript sequence, and cuffcomapare software was used to compare the variable splice sequence of this project with the reference transcript sequence (gff), using ASTALAVISTA software to analyse the variable splicing event. Such sequences were defined as unigenes, which were then aligned with the NR, GO, KEGG, eggNOG, and Swiss-Prot databases.

### Differential gene expression and gene enrichment analysis

To analyze differential gene expression, candidate genes were identified with DESeq software [38, 39] in each comparison. Transcripts that exhibited two-fold or above were considered as differentially expressed, and the *P*-value threshold was set to 0.05. Enrichment patterns were clustered by pheatmap software using a complete linkage method. The unigenes were aligned to the GO and KEGG databases to predict the possible functions and metabolic pathways involved. GO terms were assigned by the Blast2GO program [40]. The KEGG Automatic Annotation Server (KAAS) was used for pathway annotation. The database searches were performed using BLASTX [41] with a cut-off *E*-value of 10^−5^. The enrichment of terms in the different treatments was further analyzed (*P* < 0.05).

### Amino acids detection

The samples for the analysis of amino acids were the same as those used in transcriptome sequencing. The pseudostem samples (0.5 g) with three biological replicates were treated with 5 mL of 10 % acetic acid and grinding on ice. The extractions were centrifuged at 12,000 rpm for 20 min. The filtered liquid was collected to enable amino acid detection. All of the extracted filtrates were filtered through 0.45 μm membranes before being measured. Amino acids were quantified using an amino acid analyzer (Hitachi, L-8900, Japan) with standard methods. Statistical analyses were performed using OriginPro to evaluate the statistical significance of differences between different culture conditions. Analysis was conducted with three technical replicates, and error bars (± SEM) were shown for independent experiments. Significances were calculated using an ANOVA approach, and the Tukey test was applied to determine differences between treated and untreated samples. *P*-value < 0.05 was considered significant.

## Data Availability

RNA-seq raw data were deposited in the SRA database of NCBI with accession number PRJNA504406.

## Abbreviations

N: Nitrogen
NUE: nitrogen use efficiency
RNA-sec: Ribonucleic acid-sequencing
GOGAT: glutamate synthase
GS: glutamine synthetase
DEG: differentially expressed gene
GO: Gene Ontology
KEGG: the Kyoto Encyclopedia of Genes and Genomes
eggNOG: evolutionary genealogy of genes:Non-supervised Orthologous Groups
NR: nitrate reductase
AsuS: argininosuccinate synthase
ASL: argininosuccinate lyase
GDH: glutamate dehydrogenase
AMT: ammonium transporter
TCA: tricarboxylic acid cycle
PCR: polymerase chain reaction
KAAS: KEGG Automatic Annotation Server
